# Qualitative interviews of patients with methamphetamine-associated pulmonary hypertension: Understanding the unmet medical need from the patient lens^1^

**DOI:** 10.1016/j.jhlto.2026.100549

**Published:** 2026-03-30

**Authors:** Lana Melendres-Groves, Vinicio De Jesus Perez, Anjali Vaidya, Hyein G. Lee, Marinella Sandros, David Lopez, Ankita Adhia, Natalie Gearhart, Michelle Cho, John F. Kingrey

**Affiliations:** a University of New Mexico Health Sciences, Albuquerque, NM; b Stanford Medicine, Stanford, CA; c Temple Health, Philadelphia, PA; d Putnam Associates, New York, NY; e Johnson and Johnson, Titusville, NJ; f Integris Health, Oklahoma City, OK

**Keywords:** Pulmonary arterial hypertension, Substance use, Methamphetamine, Methamphetamine-associated pulmonary arterial hypertension, Qualitative research, Patient experience

## Abstract

The rise of methamphetamine-associated pulmonary arterial hypertension in the U.S. highlights a need for appropriate and effective management strategies. The complexities of Methamphetamine-Associated Pulmonary Arterial Hypertension are magnified by socioeconomic and health disparities associated with substance use disorders. This study asked: what are the experiences, barriers, and unmet needs of patients living with methamphetamine-associated pulmonary arterial hypertension?

Structured interviews were conducted with 20 U.S. patients living with methamphetamine-associated pulmonary arterial hypertension. Interviews explored methamphetamine use patterns, circumstances of Pulmonary Arterial Hypertension diagnosis, treatment experiences, barriers to care, and perceptions of compassion and education during management. Participants were geographically diverse; most were female and White, with nearly half over 50 years old.

Patients report having used methamphetamine almost daily, typically by inhalation; more than one-third reported use for over 10 years. At diagnosis, 60% of patients were still actively using, and half of these patients discontinued use within 1 year of diagnosis. Initial diagnosis commonly occurred in emergency departments, and delays in treatment were frequent, often related to lack of insurance or negative experiences with physicians. Patients described inadequate education and limited collaboration with substance use specialists. Compassionate care was perceived as lacking, particularly among those treated outside pulmonary hypertension specialty centers.

This first-of-its-kind patient-centered study of methamphetamine-associated pulmonary arterial hypertension highlights major unmet needs in timely diagnosis, access to therapy, and provision of compassionate, informed care. Findings underscore the urgency of increasing awareness, strengthening education for both patients and providers, and integrating substance use support into pulmonary hypertension management.

## Background

Pulmonary arterial hypertension (PAH) is a progressive and potentially life-threatening disease characterized by elevated pulmonary vascular resistance, associated with reduced quality of life and increased morbidity and mortality.^1^, ^2^, ^3^, ^4^ Among the recognized risk factors for PAH is the use of drugs and toxins, including methamphetamine.^5^, ^6^ Drug and toxin-associated PAH, such as Methamphetamine-associated PAH (Meth-APAH), has been recognized as a subtype of PAH since the 6th World Symposium on Pulmonary Hypertension^7^ and has gained growing attention amid the rising prevalence of methamphetamine use in the United States (US) and globally. Meth-APAH represents a growing and pressing public health crisis with significant implications for both patients and healthcare systems.^8^

The prevalence of methamphetamine use has risen dramatically in recent years, with mortality associated with methamphetamine overdose more than quadrupling between 2011 and 2018 in the US.^9^ The burden of Meth-APAH is growing in parallel, not only in US regions considered endemic, such as the West but also across the Midwest and the South.^10^, ^11^ The rise of Meth-APAH disproportionately affects populations with socioeconomic vulnerabilities, including individuals experiencing homelessness, poverty, or co-occurring substance use disorders.^9^, ^12^

Methamphetamine is a potent sympathomimetic agent that induces pulmonary vascular remodeling, endothelial dysfunction, and increased pulmonary vascular resistance, which may contribute to the development of PAH.^8^, ^13^ Clinically, Meth-APAH has a similar presentation to idiopathic PAH with nonspecific symptoms such as dyspnea, chest pain, dizziness, lower extremity edema or reduced exercise tolerance, which may be initially attributed to acute methamphetamine intoxication rather than underlying PAH. This overlap in symptomatology can frequently delay diagnosis, contributing to worse outcomes.^10^

The interplay between methamphetamine use and social determinants of health further complicates the management of Meth-APAH. Patients with active or past methamphetamine use often face barriers to care stemming from societal stigma associated with substance use disorders, limited access to specialized medical attention, and competing priorities related to socioeconomic instability such as housing and addiction management.^14^ These socioeconomic challenges amplify the burden of Meth-APAH and pose significant hurdles for patients and healthcare providers managing Meth-APAH.

As there remains limited understanding of the patient perspective and experiences among this population, this study asked what are the experiences, barriers, and unmet needs of patients living with Meth-APAH. To address, we conducted a first-of-its-kind qualitative research to hear and share the stories of 20 patients with Meth-APAH across the US. By bringing real-world experiences of patients to light, this study aims to support the development of best practices in compassionate, patient-centered care pathways for Meth-APAH that improve both clinical and quality-of-life outcomes.

## Methods

### Participants

Twenty participants were recruited through a US-wide patient recruitment vendor specializing in engaging with patients with rare diseases using existing patient panels. Participants were deemed eligible for this study if they were adults (aged 18 or older) with a history of methamphetamine use, a documented diagnosis of PAH at least 12 months prior to the time of the interview, and had disclosed their methamphetamine use with their treating physician, as assessed by their self-reported responses to a structured questionnaire. No independent verification of medical history was conducted. Demographic characteristics of the participants are summarized in the Results section.

This study did not undergo Institutional Review Board approval, as it was a voluntary, non-interventional, interview-based study. Informed consent was obtained from all participants, both verbally and in writing. Participants were compensated for their time. Measures were taken to minimize risk and ensure strict confidentiality. All interview data were de-identified prior to analysis, and participants were assigned unique study identifiers during coding to support analysis. Audio recording and transcripts were stored on secure, access-restricted systems. All findings are reported in aggregate to protect participant confidentiality.

### Method and design

Structured interviews lasting approximately 45 minuets were conducted via teleconference between July and August 2024. The interviews followed a guide developed for this study in collaboration with all authors, which include physicians experienced in Meth-APAH care. The guide included a mix of open-ended questions and predefined themes to ensure consistency while allowing participants to share their experiences. Key themes included methamphetamine use behavior and history, path to diagnosis and PAH treatment, barriers to accessing care, and patient perspectives on managing Meth-APAH.

### Analysis

All interview recordings were transcribed verbatim and analyzed using thematic analysis to identify patterns and recurring themes. All coding was conducted manually by three members of the research team who were trained and have extensive experience in qualitative patient research using a codebook developed based on the research guide and iteratively refined based on findings. The coders were not involved in the clinical care of participants and had no prior relationship with them. Coding was performed independently, followed by discussions to reconcile differences and refine the final thematic framework. Transcripts and coded data were de-identified prior to analysis to maintain participant confidentiality. Where comparisons are made in the findings, statistical testing was carried out to confirm significance, and details can be found in the corresponding figure text.

## Results

### Study participant characteristics

The demographic characteristics of the participants resemble those reported in previous publications (Table 1).^10^, ^11^ Most participants resided in the West and the South, which aligns with previous studies on the distribution of methamphetamine use and Meth-APAH populations in the US. Participants were predominantly female, largely 35 or older, and primarily White.

**Table 1:** Demographics of Patients With Meth-PAH in This Study

	Patients with Meth-APAH (*N* = 20)
*Current Age, % (N)*	
26-34	5% (1)
35-49	50% (10)
50+	45% (9)
*Gender, % (N)*	
Female	90% (18)
Male	10% (2)
*Race, % (N)*	
Black	10% (2)
Multiracial*	5% (1)
Native American	10% (2)
White	75% (15)
*Geographical Location, % (N)*	
Midwest	20% (4)
Northeast	10% (2)
South	30% (6)
West	40% (8)
*Educational Attainment, % (N)*	
Did Not Finish High School	10% (2)
High School Diploma/GED	60% (12)
Associate’s Degree	15% (3)
Bachelor’s Degree	15% (3)
*Employment Status*^a^*, % (N)*	
Full-Time	50% (10)
Part-Time	15% (3)
Unemployed	35% (7)
*Health Insurance Status*^a^*, % (N)*	
Commercial	30% (6)
Medicaid	45% (9)
Medicare	10% (2)
Uninsured	15% (3)

Abbreviation: GED, General Educational Development Test.

* Patient identifies as Black and White.

a at time of diagnosis.

Most had received a high school diploma or General Education Development, and a smaller proportion had earned an associate degree or above. Half of the participants were working full-time at the time of PAH diagnosis. Health insurance coverage was varied, with the largest proportion of participants covered by Medicaid at the time of PAH diagnosis.

### Methamphetamine use history

**Figure 1:**
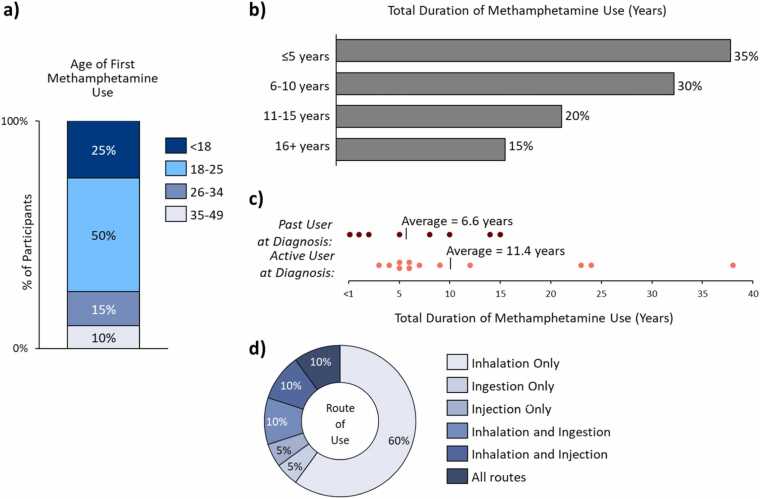
*Methamphetamine use behaviors among patients with Meth-APAH.* Methamphetamine use behavior of study participants, including age of first use, duration of use, and route of use. (a) Age of first methamphetamine use among study participants; (b) duration of active use among study participants; (c) duration of use, by use status at diagnosis, (d) route of methamphetamine usage over the course of use among study participants. *Abbreviation:* Meth-APAH, Methamphetamine-associated Pulmonary Arterial Hypertension.

Three-quarters of the participants started methamphetamine use before the age of 25, with a quarter of beginning use before the age of 18 (Figure 1a). The most common reasons for initiating methamphetamine use were social or relationship pressures and the desire to seek emotional or physical pain relief.

The majority of participants (70%, *N* = 14/20 patients) used methamphetamine daily during their periods of active use. The median duration of use was 6.5 months across participants, and the total duration of use overall varied greatly (Figure 1b). Participants who had ceased methamphetamine use prior to PAH diagnosis (“past user”) were likely to have used for a shorter duration compared to those who were diagnosed with PAH during active use (“active user”) (Figure 1c).

Routes of administration (RoA) of methamphetamine also varied. Though 90% initially used methamphetamine via inhalation, over time, 30% escalated to multiple routes, such as ingestion or injection, in addition to inhalation; a minority started with multiple RoAs from first use (Figure 1d).

**Figure 2:**
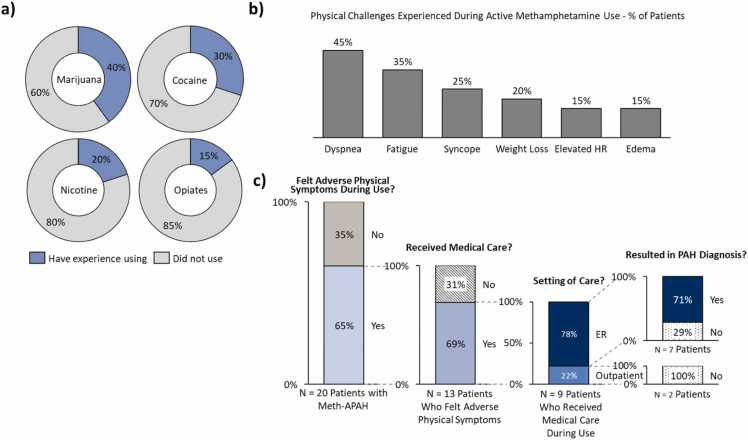
*Methamphetamine use behaviors of patients with Meth-APAH.* Methamphetamine use behavior - including other substances used and duration of methamphetamine use - and physical challenges experienced during the time of methamphetamine use. (a) Other substances most frequently mentioned as being used during time of active methamphetamine use by study participants; (b) Physical challenges experienced by study participants during active methamphetamine use; (c) Medical attention and PAH diagnosis received during the time of active methamphetamine use. *Abbreviations:* HR, Heart Rate; Meth-APAH, Methamphetamine-associated Pulmonary Arterial Hypertension.

Concurrent use of other illicit substances during the periods of active methamphetamine use was common, with 75% of participants reporting other substance use. The most frequent substances were marijuana, cocaine, nicotine, and opiates (Figure 2a). Participants recall using other substances to mitigate methamphetamine's stimulatory effects. For example, patients said that marijuana was used to aid sleep while nicotine evoked the feeling of improved breathing.

Nearly two-thirds of participants (65%, *N* = 13/20 patients) had experienced adverse physical challenges during their active methamphetamine use, which frequently overlapped with symptoms of PAH. Most common were dyspnea, fatigue, and syncope (Figure 2b). Less frequent were weight loss, elevated heart rate, and edema. Some participants adjusted their methamphetamine use in response through de-escalating their methamphetamine use (i.e., using less frequently or ceasing IV use) or using other substances to relieve adverse sensations of methamphetamine use. The median delay in diagnosis from experiencing these physical challenges was 14 months though this varied greatly; even across patients who experienced visiting the ER due to syncope, some participants were soon after diagnosed with Meth-APAH while one participant was diagnosed 14 years after.

Many of the participants who experienced adverse physical challenges received medical care at the time (Figure 2c). Of these, the majority sought care at emergency rooms (ERs). Over half of these participants (57%; *N* = 4/7 patients) visited voluntarily with symptoms like dyspnea, the rest (43%; *N* = 3/7 patients who received ER care during active use) were involuntarily hospitalized following episodes of syncope. Most of the patients who received medical attention at the ER during active use were diagnosed with PAH at their visit.

Of the participants who sought care at an outpatient setting, dyspnea was the most common symptom of concern. None of these participants received a diagnosis of PAH at the time, as their symptoms were attributed to other conditions such as obesity and drug use (Figure 2c).

### Path to PAH diagnosis

**Figure 3:**
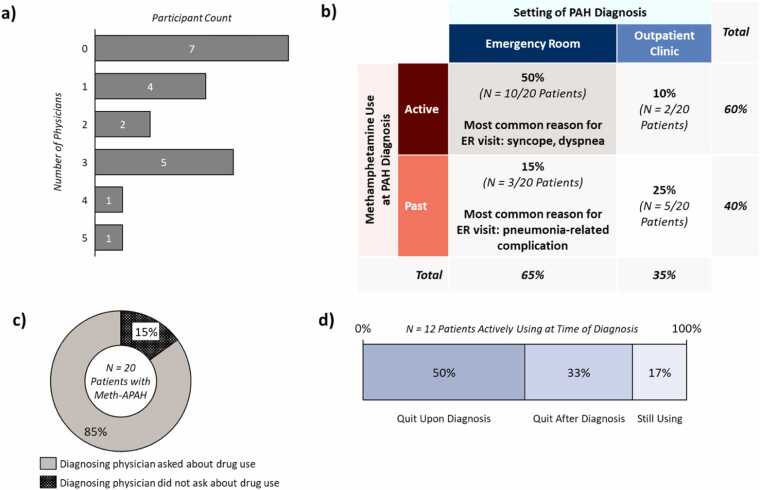
*Path to Meth-APAH Diagnosis.* Details of the PAH diagnostic journey and changes in methamphetamine use as a result of the PAH diagnosis of 20 patients of Meth-APAH interviewed in this study. (a) Number of physicians seen by patients about physical challenges experienced before seeing the physician who diagnosed Meth-APAH; (b) Setting of PAH diagnosis and methamphetamine use status at time of PAH diagnosis; (c) Percentage of patients who were asked about their methamphetamine use history at time of diagnosis; (d) Percentage of patients actively using methamphetamine at diagnosis who quit use upon, after, or did not quit use at time of interviews. *Abbreviations:* ER, Emergency Room, Meth-APAH, Methamphetamine-associated PAH, PAH, Pulmonary Arterial Hypertension.

Most participants had seen multiple physicians prior to their Meth-APAH diagnosis about the physical challenges they experienced (Figure 3a). Most participants were diagnosed following a visit to the ER (Figure 3b). Among those diagnosed in the ER, the majority were active users at the time of diagnosis; among those diagnosed in the outpatient setting, the majority had ceased use prior to diagnosis. Similarly, participants who were active users at time of PAH diagnosis were more likely to be diagnosed following an ER visit, while participants who had ceased use before diagnosis were more likely to be diagnosed in the outpatient setting.

Among patients who were diagnosed with PAH following a visit to the ER, the most frequent reasons for ER admission differed by methamphetamine use status at time of diagnosis. For those who were active users, 30% (*N* = 3/10 patients) recall being admitted due to symptoms associated with severe PAH such as syncope and dyspnea. The most frequent reason for ER admission among past users diagnosed following an ER visit was pneumonia-related complications.

Most but not all recall being asked about their past and current drug use by their diagnosing physician (Figure 3c). All participants diagnosed during their ER visit recall being asked, but nearly half (43%; *N* = 3/7 patients diagnosed in an outpatient clinic) of participants diagnosed in an outpatient clinic state they were not asked about their drug use history by the diagnosing physician; two of these participants voluntarily disclosed their methamphetamine use years after diagnosis, while one disclosed at their first follow-up appointment after diagnosis.

Most of the participants who were asked about their drug use (88%, *N* = 15/17 patients) say they truthfully and willingly disclosed. Less than half of the patients who were asked about their drug use (47%, *N* = 8/17 patients) were assessed for ongoing drug use with toxicology screens.

Among participants who were active users at the time of their PAH diagnosis, half quit immediately following their diagnosis (Figure 3d) due to the emotional shock of the diagnosis and stern advice from their physicians. They note they had not fully understood the seriousness of their health until their PAH diagnosis. A third of these participants continued to use after diagnosis but eventually stopped; two participants quit after receiving an ultimatum from their physician that treatment escalation would be withheld if drug use continued, while two other participants quit as they “felt ready to stop using drugs.” At the time of the interviews, two participants were still using methamphetamine, expressing that they will eventually quit if their PAH symptoms worsened.

None of the participants who quit upon diagnosis were referred to formal support programs. Among those who eventually stopped following their diagnosis, only half were referred to support programs. Both participants who continued to use methamphetamine at the time of the interviews were referred to support programs but found them ineffective.

### Meth-APAH treatment regimens

**Fig. 4:**
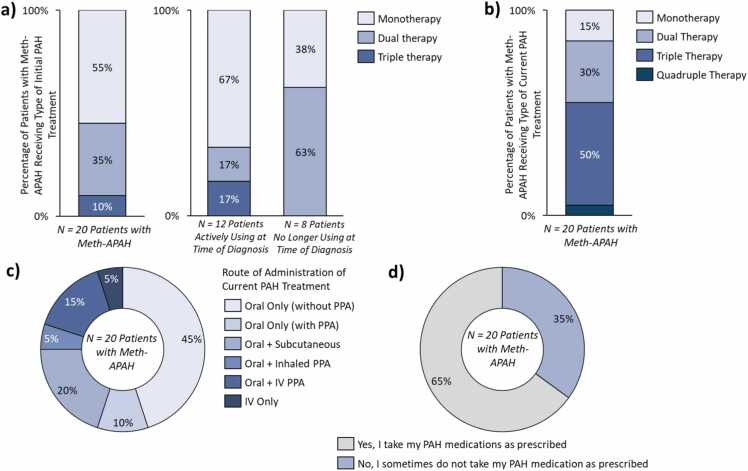
*Prescribed PAH treatments of patients with Meth-APAH.* Treatment regimens and reported treatment adherence of 20 patients with Meth-APAH interviewed in this study. (a) Distribution of initial PAH treatment type prescribed at diagnosis overall and by methamphetamine use status at PAH diagnosis; (b) distribution of current PAH treatment type prescribed at time of interviews; (c) distribution of route of administrations of medications in current PAH treatment at time of interviews; (d) patient-reported adherence to PAH medication at time of interviews. *Abbreviations:* IV, Intravenous, Meth-APAH, Methamphetamine-associated PAH, PAH, Pulmonary Arterial Hypertension, PPA, Prostacyclin Pathway Agent.

Participants were most commonly initiated on monotherapy for PAH (Figure 4a). Differences in initial PAH treatment approach were seen based on methamphetamine use at PAH diagnosis; for active users at diagnosis, the majority were initiated on monotherapy, while past users were more likely to be prescribed dual therapy. Initiation with triple therapy was higher in active users compared to past users.

Most participants had changed their PAH regimen since initiation (Figure 4b). Majority escalated treatment (55%, *N* = 11/20 patients); of these, most escalated from monotherapy to dual or triple therapy (73%, *N* = 8/11 patients). Some participants maintained the same number of drugs in their regimen but had changed the specific drugs (25%, *N* = 5/20 patients).

The most common RoA of current PAH treatments prescribed were oral-only (Figure 4c). Half of the participants had conversations with their treating physician about the routes of methamphetamine usage they had used, and fewer (10%, *N* = 2/20 patients) had discussed how their history and route of methamphetamine use may influence treatment choice. Few participants who are currently receiving parenteral PAH treatment had previously used methamphetamines intravenously (18%; *N* = 2/11 patients).

### Treatment adherence

Most participants stated they are adherent to their PAH treatment (Figure 4d). Adherent participants credited the perceived benefits of the treatments and proactive providers who checked in regularly. Among partially adherent participants, barriers to adherence included forgetfulness, complexity of some PAH medications, difficulties tracking refills, and side effects such as infusion site pain.

### Unmet needs felt by patients

**Figure 5:**
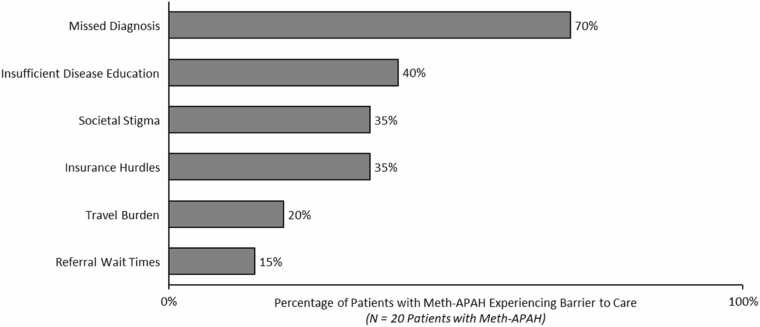
*Unmet needs experienced by patients with Meth-APAH.* Barriers and challenges in PAH diagnosis and management as experienced by 20 patients with Meth-APAH interviewed in this study. Percentage of patients with Meth-APAH who recalled experiences with barriers to PAH care during their Meth-APAH patient journey. *Abbreviation:* Meth-APAH, Methamphetamine-associated PAH.

The most frequent challenges mentioned by participants were missed diagnoses, insufficient disease education, societal stigma, and insurance hurdles (Figure 5). Delays in diagnosis were nearly universal, with 95% (*N* = 19/20 patients) of participants reporting delays of weeks to years since the start of their PAH symptoms.

The majority of participants saw multiple healthcare providers, including cardiologists and pulmonologists, who misdiagnosed their PAH for conditions such as anxiety, obesity, or asthma. Many (35%, *N* = 7/20 patients) delayed seeking medical attention until symptoms became severe due to societal stigma; they recalled their previous experiences of feeling judged or dismissed, which eroded their trust in medical providers.

Many participants were dissatisfied with the education they received about PAH, with most (75%; *N* = 6/8 patients) still feeling that they do not know enough about PAH. Notably, all participants who were seeing a non-PAH-specialist for the care believe they were insufficiently educated about PAH (*N* = 4/4 patients), and only one (24%; *N* = 1/4 patients) was taught about the link between methamphetamine use and PAH. On the other hand, most participants who reported receiving care from a PAH specialist state they were sufficiently educated about their disease (79%; *N* = 11/14 patients) and that their physician educated them about the link between methamphetamine use and PAH (71%; *N* = 10/14 patients). Two participants were unsure if the physician managing their PAH was a PAH specialist.

Additional challenges experienced by patients included insurance hurdles to receive PAH treatment, travel burden to PAH specialty centers, and referral wait times, all which contributed to delayed treatment initiation. Among patients who were uninsured at diagnosis, they experienced an average of 19 months between their diagnosis and their first treatment. Participants who experienced referral wait times to see a PAH specialist reported waiting 6-8 months for their appointment.

## Discussion

This first-of-its-kind qualitative study captures the patient perspectives and experiences with managing Meth-APAH, offering novel insights into methamphetamine use history and behavior, path to Meth-APAH diagnosis, prescribed Meth-APAH treatments, and key hurdles faced by patients with Meth-APAH. The results highlight delayed diagnoses, inadequate patient education, socioeconomic obstacles, and the critical influence of provider compassion and stigma on patient engagement. Together, these insights underscore major opportunities to improve the care of patients with Meth-APAH.

The patient demographics in this study, as well as other cohort and claims research on Meth-APAH patients, demonstrate the multifactorial challenges patients with Meth-APAH face daily.^10^, ^12^ Among the participants of this study, many were unemployed and some were uninsured at diagnosis. In addition to managing socioeconomic instability commonly associated with people with past or ongoing substance use disorders, patients with Meth-APAH note struggling with insurance hurdles and travel burden in managing their disease.

Interestingly, the total duration of methamphetamine use (ranging from less than 1 year to 38 years) as well as the RoA of use was found to vary greatly within the study cohort. This observation highlights the ongoing knowledge gap in the field of how methamphetamine use duration and RoA correlate to the likelihood and time to develop PAH.

The high rate of delayed diagnoses patients with Meth-APAH faced is notable. Most patients (*N* = 13/20 patients) recalled seeing multiple providers who misattributed their PAH symptoms to conditions such as anxiety, asthma, obesity, or to their methamphetamine use. Combined with the higher rates of PAH diagnoses among active methamphetamine users in the ER setting observed in this study, our findings align with previous observations of patients with Meth-APAH being diagnosed at a worse functional state than other PAH patients.^10^, ^12^ This suggests focused education of frontline healthcare workers may be most effective in improving earlier diagnoses of Meth-APAH. Syncope in methamphetamine users emerges as a potential diagnostic signal in this context.

None of the patients knew about the connection between methamphetamine use and PAH or recognized their symptoms as attributable to PAH. Broader educational initiatives among newly diagnosed PAH patients and methamphetamine users, as well as public health campaigns, could help improve patient-driven suspicion of Meth-APAH.

The study also revealed significant gaps in patient education post-diagnosis. Nearly half of the participants reported the education they received from their care team about PAH was insufficient, and some state they continue to feel ill-informed about PAH at the time of the interviews. Lack of education was especially notable in patients who were not receiving care from a PAH specialist. It is notable that during participant recruitment, 22% of potential participants were excluded as they had not discussed their methamphetamine use with their PAH provider.

Participants highlighted several challenges in accessing care for their Meth-APAH, including insurance-related obstacles, travel burden to seek care, and extended referral wait times. Additionally, none of the patients who were actively using at the time of diagnosis received referrals to substance use cessation support programs, highlighting a gap in the integration of supplemental addiction support within the care pathway. Given the clinical complexities and progressive nature of Meth-APAH, addressing these fundamental barriers and gaps in care can help alleviate the overall burden and improve outcomes of Meth-APAH management in patients.

Lastly, compassionate provider attitude emerged as a crucial factor in improving patient experience, engagement, and adherence to treatment. Adherent patients consistently attributed their adherent behavior to providers who took the time to educate, answer questions, and proactively check in, which contributed to patients feeling more open to talk about their past or ongoing drug use and to accept help. The experiences of patients in this study mirror the societal stigma and resulting aversion of healthcare that many people with substance use disorders face.^15^, ^16^ Educating providers on ways to better build trust, address stigma, and support patients with empathy can have a profound impact on treatment adherence and outcomes in Meth-APAH.

The findings of this study should be interpreted in the context of some limitations. First, the sample size of 20 patients with Meth-APAH may limit generalizability. Compared to the broader U.S. Meth-APAH population, the sample overrepresented women and those with higher educational attainment.^10^, ^11^ Geographic distribution was weighted toward the West and South, potentially limiting insights into regional differences in care. Furthermore, the qualitative nature of this research relies on self-reported data, which may introduce recall bias.

## Disclosure statement

L.M.-G.: Consulting for Johnson & Johnson**.** V.D.J.P.: Consulting for Johnson & Johnson. A.V.: Consulting for Johnson & Johnson, Merck, United Therapeutics. M.S., D.L., A.A., N.G., and M.C. are employees of Johnson and Johnson. H.G.L. has no conflicts to disclose. Research supported by Johnson and Johnson.
